# Age-Related Cancer-Associated Microbiota Potentially Promotes Oral Squamous Cell Cancer Tumorigenesis by Distinct Mechanisms

**DOI:** 10.3389/fmicb.2022.852566

**Published:** 2022-04-15

**Authors:** Zhen Zhang, Qiang Feng, Meihui Li, Zhihui Li, Qin Xu, Xinhua Pan, Wantao Chen

**Affiliations:** ^1^Department of Oral and Maxillofacial-Head & Neck Oncology, Shanghai Ninth People’s Hospital, Shanghai Jiao Tong University School of Medicine, Shanghai, China; ^2^College of Stomatology, Shanghai Jiao Tong University, Shanghai, China; ^3^National Center for Stomatology, Shanghai, China; ^4^National Clinical Research Center for Oral Diseases, Shanghai, China; ^5^Shanghai Key Laboratory of Stomatology, Shanghai, China; ^6^Department of Human Microbiome, School and Hospital of Stomatology, Cheeloo College of Medicine, Shandong University, Jinan, China; ^7^Shandong Key Laboratory of Oral Tissue Regeneration & Shandong Engineering Laboratory for Dental Materials and Oral Tissue Regeneration, Jinan, China

**Keywords:** 16S, OSCC, microbiota, age-related, cross sectional study

## Abstract

The oral squamous cell cancer (OSCC) incidence in young patients has increased since the end of the last century; however, the underlying mechanism is still unclear. Oral microbiota dysbiosis was proven to be a tumorigenesis factor, and we propose that there is a distinct bacterial composition in young patients that facilitates the progression of OSCC. Twenty elderly (>60 years old) and 20 young (<50 years old) subjects were included in this study. OSCC tissue was collected during surgery, sent for 16S rDNA sequencing and analyzed by the QIIME 2 pipeline. The results showed that *Ralstonia*, *Prevotella*, and *Ochrobactrum* were significantly enriched in younger OSCC tissue microbiota, while *Pedobacter* was more abundant in elderly OSCC tissues. *Fusobacterium* had high relative abundance in both cohorts. At the phylum level, Proteobacteria was the dominant taxon in all samples. The functional study showed that there were significant differences in the taxa abundance from metabolic and signaling pathways. The results indicated that the microbiota of younger OSCC tissues differed from that of elderly OSCC tissues by both taxon composition and function, which partially explains the distinct roles of bacteria during tumorigenesis in these two cohorts. These findings provide insights into different mechanisms of the microbiota-cancer relationship with regard to aging.

## Introduction

Oral squamous cell cancer (OSCC) normally occurs in elderly subjects between 50 and 70 years (Sarode et al., 2020) and poses a serious threat to patients’ health and quality of life. However, since the 1990s, the incidence of oral cancer in people younger than 50 years old has been continuously rising (Tota et al., 2017). Clinical studies showed that young oral cancer subjects had higher malignant levels than elderly subjects, and they were more vulnerable to local relapse and lymph node metastasis; therefore, aggressive treatment strategies were conducted (Sarkaria and Harari, 1994; Goldstein and Irish, 2005). Hence, determining the unique pathogenesis of OSCC in young subjects is vital for performing effective interventions and improving survival rate.

Currently, research on the pathogenesis of young OSCC patients is not sufficient to explain the rise in incidence. Studies have shown that classic carcinogens, such as smoking, drinking and HPV infection, are not the main reasons for the rise in incidence as young subjects have short period of time to be exposed to those carcinogens (Sarode et al., 2021). The genomic studies showed that mutations in young and elderly OSCC patients were similar, which were mainly in the genes such as *TP53, CDKN2A, NOTCH1, CASP8, FAT1, PIK3CA*, and *MLL2* (Kandoth et al., 2013; Pickering et al., 2014), but study also reported OSCC patients with younger age appeared to have an attenuated immune response due to lower load of mutations (Maroun et al., 2021), and *EGFR* was reported to have higher rate of amplification in young patients (Satgunaseelan et al., 2021). However, the mechanism underlying young OSCC incidence elevating cannot be merely explained by genomic alteration. Recent studies showed that the occurrence of OSCC was related to dysbiosis of some microbes other than HPV, since they have various carcinogenic potentials through different mechanisms (Sun et al., 2020; Tuominen and Rautava, 2021). Systematic diseases, including cancers, are not only related to single pathogens but also affected by the microbiota (Kau et al., 2011). A large number of various bacterial species have been found in tumor tissue, mainly in tumor cells and macrophages of the tumor microenvironment (Nejman et al., 2020). Pathogenic bacteria in the oral cavity provoke or aggravate inflammation, promote cell proliferation and inhibit cell apoptosis through metabolic activities (Hooper et al., 2009). Studies have also shown that compared to healthy subjects, the number of anaerobes were significantly increased in the microbiota of the OSCC oral cavity, including *Porphyromonas gingivalis*, and *Fusobacterium*, while *Streptococcus* and *Rothia* were sharply decreased (Hooper et al., 2006). The dysbiosis of microbiota is firmly related to the development of OSCC.

There is a dynamic change in microbiota composition as age increases, with an increase in alpha diversity and a drop in beta diversity, which lead to differences in immune status, hormone and metabolism levels. The current study focused on the compositional and functional differences between the microbiota of young and elderly OSCC subjects and aimed to explain the role of distinct microbiota in cancer. The findings further explain the mechanism of increased incidence of OSCC in young subjects and provide potential targets for clinical research.

## Materials and Methods

### Ethics Statement

This study was approved by the Institutional Review Board of Ninth People’s Hospital, Shanghai Jiao Tong University School of Medicine. All methods were performed according to relevant guidelines and protocols, including any relevant details. Written informed consent was obtained from each participant.

### Sample Collection

Samples of the current study were collected from the sharing platform for the tissue sample and bioinformatics database of oral maxillofacial tumors, the demographics was well recorded and Chi-square test was performed to identify potential confounders (Table 1), germ free procedures and operating environment were ensured to avoid possible contamination. The enrolled patients were assigned to young and elderly groups by age, and subjects with smoking or drinking history were excluded. Tongue cancer tissue samples were collected immediately after surgery, and all samples were stored at −80°C within 20 min for further use. Total genomic DNA of the tumor samples was extracted using the QIAGEN QIAamp DNA mini kit (Qiagen, Hilden, Germany) according to the manufacturer’s protocol. The concentration and purity of the DNA was assessed by a Nanodrop 2000 ultramicro spectrophotometer (NanoDrop Technologies, Wilmington, DE, United States).

**TABLE 1:** Demographic data of subjects enrolled in the study.

Variable	Younger group (*n* = 20)	Elder group (*n* = 20)	*p*-Value (χ^2^ test)
Age, years			
Average (range)	37.55 (26–46)	65.6 (59–80)	
Gender (F/M)			
Female	15	6	0.01131
Male	5	14	
Tumor stage			
I–II	16	10	0.09742
III–IV	4	10	
Pathologic stage			
I	5	9	0.6193
II	11	11	
Lymph node metastasis			
Yes	7	10	0.5224
No	13	10	
Alcohol			
Yes	0	0	NA
No	20	20	
Tobacco			
Yes	0	0	NA
No	20	20	

### PCR Amplification and Sequencing

The universal primers 515F 5′-GTGCCAGCMGCCGCGG-3′ and 907R 5′-CCGTCAATTCMTTTRAGTTT-3′ were applied to capture the V4–V5 region of 16S rDNA. Sample-specific 7-bp barcodes were added into the primer sequences for multiplex sequencing. The PCR reaction system was composed of 5 μL of buffer (5×), 0.25 μL of Fast pfu DNA Polymerase (5 U/μL), 2 μL (2.5 mM) of dNTPs, 1 μL (10 μM) of each Forward and Reverse primer, 1 μL of DNA Template, and 14.75 μL of ddH_2_O.The PCR program was set as follows: 98°C for 5 min for pre denaturation; 25 cycles of 98°C for 30 s for annealing, 72°C for 30 s for extension; and 72°C for 5 min for final extension. The purification of PCR products was performed with Vazyme VAHTSTM DNA Clean Beads (Vazyme, Nanjing, China) and the quantification was performed with the Quant-iT PicoGreen DsDNA Assay Kit (Invitrogen, Carlsbad, CA, United States). Then the amplicons were pooled in equal amounts, the sequencing of 16S V4–V5 fragments was performed on an Illumina MiSeq platform (Illumina Incorporate, CA, United States) with pair-end 2 × 250 bp sequencing strategy with MiSeq Reagent Kit v3 at Shanghai Personal Biotechnology Co., Ltd. (Shanghai, China). Negative controls were set to minimize the environmental contamination.

### Bioinformatical and Statistical Analysis

The sequences were filtered by a sliding window to achieve the average quality of bases to higher than Q20. The FLASH (v1.2.7) method was used to join the 16S rRNA gene paired-end sequencing data together, and the chimaera sequence was removed by USEARCH (v10.0.240). The QIIME 2 pipeline was used for the sequence analysis and clustered into operational taxonomic units (OTUs) at a 97% threshold according to the official tutorials.^1^ Raw sequence data were demultiplexed using the demux plugin following by primers cutting with cutadapt plugin. The quality filtered sequences were denoised, merged and removed chimera with DADA2. Non-singleton OTUs were aligned with mafft and used to construct a phylogeny with fasttree2. Clusters of filtered sequences were referenced to the SILVA Release 132.

Normalization was performed to build an OTU table from the raw counts, and the same types of taxa were agglomerated at the phylum, class, order, family and genus levels. A non-parametric Wilcoxon test was used to compare the biodiversity between the two groups of data. The alpha diversity of each group was tested using Kendall’s tau and Spearman’s rank correlation coefficients. The UniFrac distance of was obtained with qiime diversity core-metrics-phylogenetic order from rarefied OTU table and used to perform beta analysis, and ANOSIM was used to analyze the differences. Co-occurrence network analysis was performed by SparCC analysis. The pseudo count value in SparCC was set to 10^–6^. The cut off of correlation coefficients was set as 70 through random matrix theory-based methods as implemented in R package RMThreshold. The co-occurrence network with nodes representing OTUs and edges representing correlations between these OTUs was constructed based on the correlation coefficient. LEfSe (Linear discriminant analysis effect size) was performed to analyze the differentially distributed taxa of different groups. Random forest analysis was applied to discriminating the samples from different groups using QIIME2 with default settings, the classification model was validated by leave-one-out strategy. Reads identified in closed reference picking were used for PICRUSt (phylogenetic investigation of communities by reconstruction of unobserved states) analysis. The graphical representation of the results was performed by STAMP.

## Results

A total of 5,181,923 raw reads were obtained from the sequencing, 4,657,023 (116,425.6 in average) filtered reads were remained for further analysis, after removing the merged and chimeric reads, a total of 4,261,032 high-quality sequences (representing 82% of the total sequences) were obtained from the oral cancer samples, with an average of 106,525.8 sequences for each subject. The average length of the sequences was 422 bp, with the maximum length reaching 441 bp and the shortest length being 97 bp. Clustering of all high-quality sequences at 97% identity resulted in 11,414 taxonomic units; on average, 8.6 phyla, 20.17 classes, 34.28 orders, 55.5 families, and 83.52 genera were distributed into each subject (Supplementary Figure 1). The composition of OTU across different taxonomic levels were shown in Supplementary Figure 2.

**FIGURE 1:**
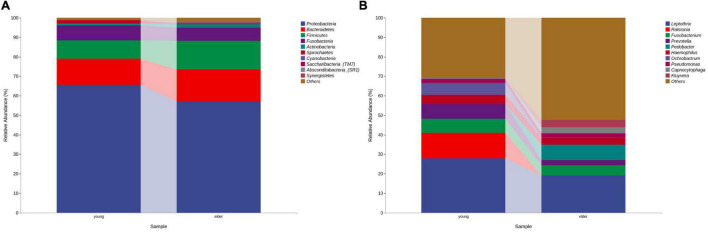
Bacterial composition at the phylum **(A)** and genus **(B)** levels by OTU analysis. The leading phyla were Proteobacteria, Bacteroidetes, Firmicutes, Fusobacteria and Actinobacteria. At the genus level, the predominant genera were *Prevotella, Leptothrix, Fusobacterium, Prevotella, Pedobacter*, etc.

**FIGURE 2:**
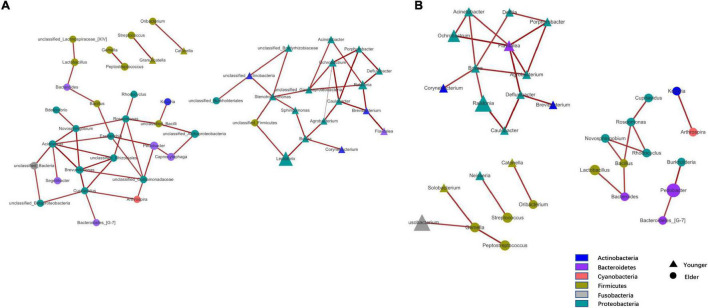
Taxa network based on unfiltered **(A)** and filtered **(B)** OTU table. Genera that were more dominant in younger group were represented by triangle and in elder group were represented by circle. Area of the shape means the relative abundance of the genera, weightiness of the line stand for the relativeness.

At the phylum level, the composition of the younger and elderly OSCC microbiomes was similar; they were mainly composed of Proteobacteria, Bacteroidetes, Firmicutes, and Fusobacteria (Figure 1A). The relative abundance of Proteobacteria in the younger and elderly groups occupied the dominant position, with proportions of 65.5 and 57.1%, respectively. The microbiota at the genus level were more differentiated than the comparison at phylum level. *Leptothrix* was the most dominant genus in both groups, but in tissue from younger OSCC patients, the relative abundances of *Ochrobactrum*, *Prevotella*, and *Ralstonia* were significantly higher, while in the elderly group, *Pedobacter* was more abundant. *Fusobacterium* had a similar position in both groups, with a relative abundance of approximately 5% (Figure 1B). The relative analysis showed that, based on the abundance of unfiltered (Figure 2A) and filtered (Figure 2B) OTU data, the genera were divided into two clusters spontaneously by their age groups, taxa under Proteobacteria were most shown.

### Microbiota Diversity Analysis

**FIGURE 3:**
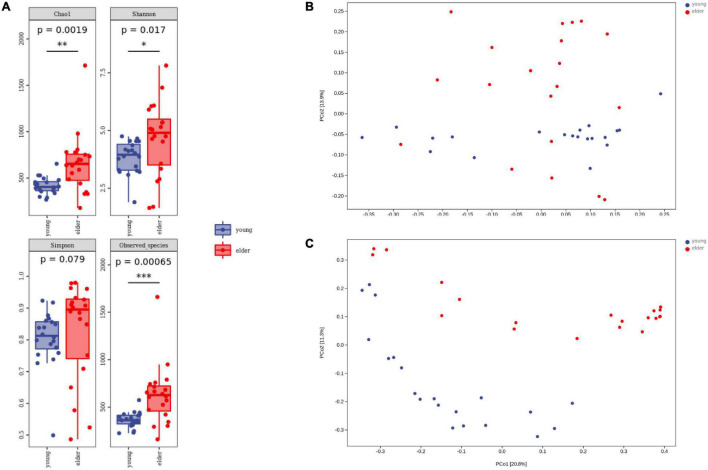
Alpha diversity analysis and beta diversity analysis. Box plots of alpha diversity indices **(A)** are shown, including Chao1, Shannon, Simpson, and Observed_species. Weighted **(B)** and unweighted **(C)** beta diversity analyses are shown on the right side. *p < 0.05, **p < 0.01, and ***p < 0.001.

To study the divergence of diversity between the two groups, alpha diversity and beta diversity analyses were performed. Measured by the number of observed OTUs, the alpha diversity indices of the Chao1, Shannon, and Simpson indices are shown (Figure 3A). It was observed that the taxa diversity of microbiota from younger OSCC tissue was significantly lower than that in elderly OSCC microbiota. The statistical significance of the comparisons was 0.0019 (Chao1), 0.017 (Shannon), 0.079 (Simpson), and 0.00065 (Observed_species). Beta diversity analysis was performed by principal coordinates analysis (PCoA), the results of weighted and unweighted analysis were shown, and the difference between the two groups was significant in both beta comparisons (Figures 3B,C). In unweighted PCoA analysis, the microbiota from the two groups was more distinct, indicating that the microbiota relative abundance between elderly and young OSCC patients was different by the amount and taxonomy of the taxa.

### Taxonomic Level Comparison

**FIGURE 4:**
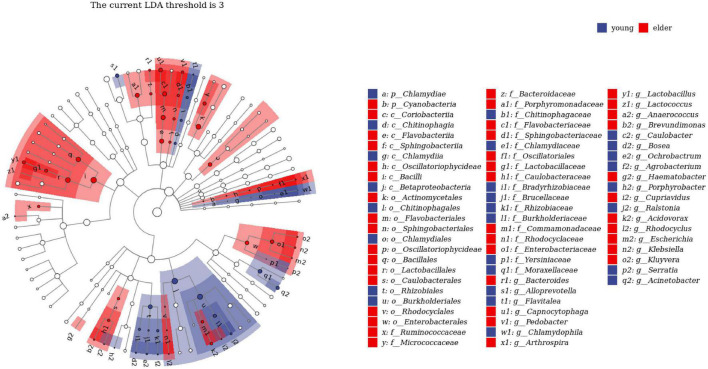
Linear discriminant analysis effect size (LEfSe) analysis between young and elderly OSCC microbiota. Red cubes represent the elderly OSCC microbiota, and blue cubes represent the young OSCC microbiota. The LDA threshold shown in the figure was set at 3.

To study the taxa that lead to divergence from different levels, LEfSe was performed to identify high-dimensional biomarkers from the comparison of the two groups (Figure 4). With LDA set as >2, significant taxa at different levels were identified. The top taxa in the younger group were *Betaproteobacteria*, *Burkholderiales*, *Ralstonia*, *Burkholderiaceae*, and *Rhizobiales*, while the most enriched taxa in the elderly group were *Enterobacteriaceae*, *Enterobacterales*, *Sphingobacteriia*, *Sphingobacteriales*, and *Pedobacter*.

**FIGURE 5:**
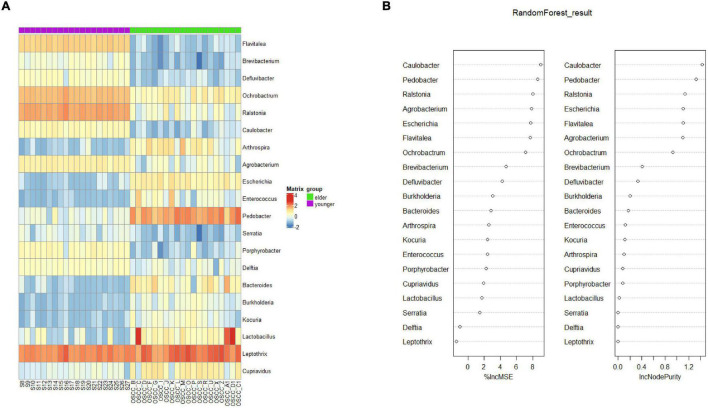
Biomarker analysis by random forest. **(A)** Biomarkers at the OTU level were selected by the random forest method. **(B)** The percentage increase in mean squared error (%lncMSE) and increase in Node Purity (lncNodePurity) of the screened biomarkers.

To determine the biomarker that differentiated the microbiota of the two groups, random forest (rf) analysis was performed at the genus level (Figure 5A). The selected biomarkers exhibited significantly different relativeness from two groups. The percentage increase in mean square error and increase in Node Purity showed the potential of the candidate biomarkers in differentiating the two groups (Figure 5B). To examine the classification model composed of the result from rf analysis, leave-one-out validation was performed, the confusion matrix was shown in Table 2, the sensitivity and specificity of the classification were 0.86 and 0.94, respectively.

**TABLE 2:** Confusion matrix constructed by classification model of random forest.

Confusion matrix (*n* = 40)		Actual class	
		
		Young	Old
**Predicted class**	**Young**	19	1
	**Old**	3	17

### Functional Prediction of Predominant Taxa From Elderly and Younger Oral Squamous Cell Cancer Microbiota

**FIGURE 6:**
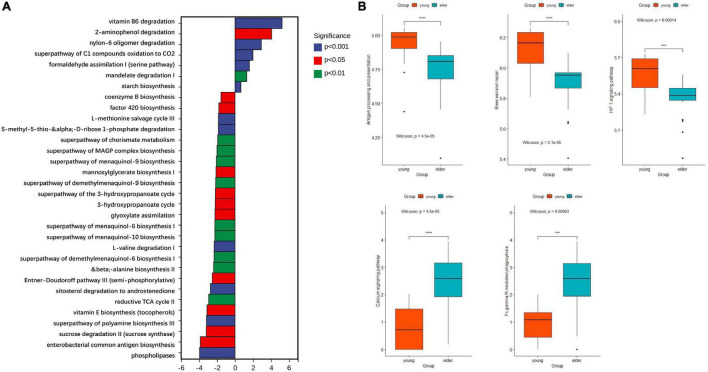
Functional prediction of microbiota by metabolic **(A)** and cancer related signaling pathways **(B)**. The top enriched metabolic pathways in the young OSCC microbiota were vitamin B6 degradation, 2-aminophenol degradation, super pathway of oxidation of C_1_ compounds to CO_2_ and formaldehyde assimilation I. ***p < 0.001 and ****p < 0.0001.

Based on the results from PICRUSt2, the functions of microbiota from different groups were predicted with 16S rDNA sequences. Their ability to affect metabolism and cancer-related signaling pathways was studied. When compared with the microbiota in the elderly OSCC group, the metabolic activities of vitamin B6 degradation, 2-aminophenol degradation, super pathway of oxidation of C_1_ compounds to CO_2_ and formaldehyde assimilation I were upregulated (Figure 6A); on the other hand, phospholipase metabolism, enterobacterial common antigen biosynthesis, sucrose degradation, polyamine biosynthesis and vitamin E biosynthesis were more enhanced in the elderly group. The research on signaling pathways showed that taxon from the two groups had different roles in pathways, such as antigen processing and presentation, base excision repair, the HIF-1 signaling pathway, the calcium signaling pathway and Fcγ R mediated phagocytosis (Figure 6B).

### Relative Analysis of Clinical Information

**FIGURE 7:**
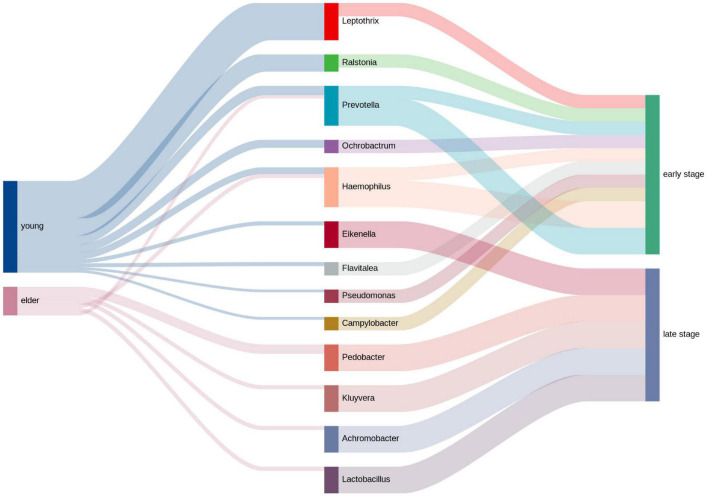
Relative analysis of taxa against tumor stage. The correlations between the top taxa in the younger and elderly OSCC microbiota are shown in the diagram. Thickness of the line of the left half represented the relative richness of the taxa, on the right half thin lines were for taxa from younger group and thick lines were for taxa from elder group.

The clinical information of the OSCC patients was collected, and the top genera from each group were extracted to calculate the relativeness with the clinical data. The top genera related to tumor stage and lymph node metastasis are shown in Figure 7. The results showed that in the young OSCC microbiota, the top genera were more related to early tumor stages, while in the elderly OSCC microbiota, the top genera were more related to late tumor stages. In the analysis of lymph node metastasis, no significant difference was observed. Significant association between the abundance of *Ochrobactrum*, *Stenotrophomonas*, and *Fastidiosipila* with the age of OSCC patients were obtained when the full dataset was used (*n* = 40) (Supplementary Figure 3).

## Discussion

It is a global issue that the cancer incidence in young people (under 50 years old) is continuously increasing (Siegel et al., 2020). Adolescent and young adult (AYA) cancers represent a unique spectrum of malignancies (Gupta et al., 2020), and the rapidly growing numbers of subjects have resulted in an epidemiological problem. In the current study, we focused on the increasing incidence of OSCC in young adults. The demographics showed that the gender distribution in the two groups is unbalanced, with more female in the younger group and more male in elder group. Since the differences in hormones and metabolisms may lead to shift of the microbiota, the confounding effects need to be considered. The underlying mechanism of young OSCC incidence involves a complex combination of changes in environmental and societal factors. When genomic studies failed to reveal the characteristics of young OSCC patients, we put our efforts into studying oral microbiota. The oral cavity is a relatively exposed environment and the starting point of the digestive tract, and it contains approximately 1,000 species of commensal microbes with crucial roles related to the health status of individuals (Sun et al., 2020). Therefore, by investigating the composition of microbiota in young and elderly OSCC tissues, we hoped to explain the differences between them and the consequences of those differences.

Humans are considered meta organisms that consist of themselves as well as various microbial communities (Greer et al., 2016). Up to trillions of individual bacterial cells have colonized the surface of the mouth, skin, airways, vagina, genitourinary tract, and gastrointestinal tract. The oral microbiota mainly belongs to the phyla Firmicutes, Actinobacteria, Bacteroidetes, Proteobacteria, Fusobacteria, and Spirochaetes (Verma et al., 2018), which was proven by our results. At the genus level, the most common genus was *Streptococcus*, followed by *Haemophilus*, *Actinomyces*, and *Prevotella* (Gao et al., 2018). Although not as well studied as the gut microbiome, some evidence has shown that there are links between increasing age and microbiota shifting, and changes in the immune system may play a role, leading to chronic inflammation in the oral cavity and increasing an individual’s susceptibility to various oral diseases (Feres et al., 2016; Belibasakis, 2018). Studies have reported that in healthy subjects, the difference in oral microbiota was not significant between young and elderly individuals, but in individuals with oral diseases, some taxa were observed to be increased in young and elderly individuals (Feres et al., 2016).

The compositional analysis of the young and elderly OSCC microbiota showed that Proteobacteria was most predominant in both groups; Proteobacteria is normally seen in the microbiota of OSCC tissue, as the acidic and anaerobic environment is suitable for the growth of members under this phylum (Ringel et al., 2015). However, at the genus level, we found that the relative abundances of *Ochrobactrum*, *Prevotella*, and *Ralstonia* were significantly higher in younger group. Species of *Ochrobactrum* are seen in prostate cancer and bladder cancer (Apisarnthanarak et al., 2005; Yu et al., 2015). They are usually observed in patients with immunological suppression (Qasimyar et al., 2014), and some members of this genus may increase resistance to antibiotics (Higgins et al., 2001). *Prevotella* has been well studied in the oral microbiota; it is the second most abundant oral genus in the mouth, exceeding 10% of the whole microbiome (Segata et al., 2012). *Prevotella* is related to poor oral hygiene and the cause of oral diseases, such as prevalent gingivitis and periodontitis (Teles et al., 2012; Larsen, 2017). The anaerobic species of *Prevotella* are responsible for chronic inflammatory processes, and the inflammatory mediators produced by them lead to disorders of fibroblasts, epithelial cells and extracellular matrix components, increasing the risk of tumorigenesis (Hoare et al., 2019). Species of *Ralstonia* are opportunistic pathogens that are related to various human diseases; they are normally seen in infections of the respiratory system (Block et al., 2013), and their high abundance is associated with persistent inflammation and the development of cancer (Sung et al., 2020). The high abundance of these genera indicates a poor status of the oral cavity microenvironment.

On the other hand, the LEfSe results indicated that *Betaproteobacteria*, *Burkholderiales*, *Ralstonia*, *Burkholderiaceae*, and *Rhizobiales* were potential biomarkers for the young OSCC microbiota, while *Enterobacteriaceae*, *Enterobacterales*, *Sphingobacteriia*, *Sphingobacteriales*, and *Pedobacter* were potential biomarkers for the elderly OSCC microbiota. The results of random forest analysis showed that *Caulobacter*, *Pedobacter*, *Ralstonia*, *Escherichia*, *Flavitalea*, and *Ochrobactrum* were effective genera in differentiating the two groups by the increase in MSE and increase in Node Purity. We examined the relationship between the biomarker and clinical information, and the results showed that the top taxa in the young OSCC microbiota were more enriched in early tumor stages, while the biomarkers for the elderly OSCC microbiota were more related to late tumor stages. Based on the compositional analysis and clinical correlation analysis of the microbiota between the two groups, we assumed that the microbiota of younger OSCC is more associated with inflammation, while the elder OSCC microbiota was more likely to help the tumorigenesis to modify the oral microenvironment.

To study the functional variance that may be caused by the compositional difference, PICRUSt2 was performed. From the comparison of the detected taxa and the functional predictions between the two groups, several metabolic pathways were found more active in the younger OSCC group. Vitamin B6 serves as an antioxidant and anti-inflammatory molecule, and its degradation is related to dysbiosis of immunity and gene expression (Bird, 2018). 2-Aminophenol has been proven to exhibit antitumor effects (Pujante-Galián et al., 2020), and the degradation of these components promotes tumorigenesis in young subjects. The super pathway of oxidation of C_1_ compounds to CO_2_ and formaldehyde assimilation I are normally seen in pathogenic bacteria (Buzolyova and Somov, 1999). KEGG analysis showed that the microbiota in OSCC tissue of young subjects may work differently in the pathways of antigen processing and presentation, base excision repair, the HIF-1 signaling pathway, the calcium signaling pathway and Fcγ R-mediated phagocytosis.

## Conclusion

Taken together, the current study revealed compositional and functional differences between the microbiota of OSCC tissues from young and elderly subjects. The results showed that *Ochrobactrum*, *Prevotella*, and *Ralstonia* were enriched in the young OSCC microbiota and may promote tumorigenesis by causing inflammation and opportunistic infection. From the view of functional prediction, the microbiota of young OSCC patients was more enriched in the degradation of antitumor components and increased oxidative stress. By analyzing the microbiome from the two groups of subjects, the current study explained the difference between the tumorigenesis of young and elderly OSCC subjects from a new point of view. There were several limitations of the current research: (1) the study had a limited sample size, and the findings from the analysis of the microbiota were not linked to pathogenesis factors; (2) 16S rDNA amplicon analysis was not accurate enough to study at optimal resolution and thus, did not allow for precisely functional predictions; and (3) proper negative control need to be set during the sample collection procedure to better avoid contamination. In future studies, whole genome shotgun strategies and cell biological experiments will be performed to research the relationship between microbiota shifts and their effects on the tumorigenesis of young subjects.

## Data Availability Statement

The data presented in the study are deposited in the SRA repository, accession number PRJNA803155.

## Ethics Statement

The studies involving human participants were reviewed and approved by the Institutional Review Board of Ninth People’s Hospital, Shanghai Jiao Tong University School of Medicine. The patients/participants provided their written informed consent to participate in this study.

## Author Contributions

WC designed the project. QF and ML provided suggestions for the project. QX, XP, and ZL collected the clinical samples. ZZ and ML performed the experiments, data statistics, and bioinformatics analysis. ZZ wrote the manuscript. WC and ZZ revised the manuscript. All authors read and approved the final manuscript.

## Conflict of Interest

The authors declare that the research was conducted in the absence of any commercial or financial relationships that could be construed as a potential conflict of interest.

## Publisher’s Note

All claims expressed in this article are solely those of the authors and do not necessarily represent those of their affiliated organizations, or those of the publisher, the editors and the reviewers. Any product that may be evaluated in this article, or claim that may be made by its manufacturer, is not guaranteed or endorsed by the publisher.

## Footnotes

1
https://docs.qiime2.org/2019.4/tutorials/

## Supplementary Material

Supplementary Figure 1 Histogram showing the reads that fell into different taxonomic levels of the bacteria by sample.

Supplementary Figure 2 Circle packing chart exhibiting the taxonomic tree of microbiota from younger and elder OSCC groups.

Supplementary Figure 3 Relative analysis of pooled genera from younger and elder OSCC groups with clinical information.
