# Supplementary figures and images for: Age-Related Cancer-Associated Microbiota Potentially Promotes Oral Squamous Cell Cancer Tumorigenesis by Distinct Mechanisms
Source: Front Microbiol. 2022 Apr 15;13:852566. doi: 10.3389/fmicb.2022.852566 (PMC9051480; doi:10.3389/fmicb.2022.852566)

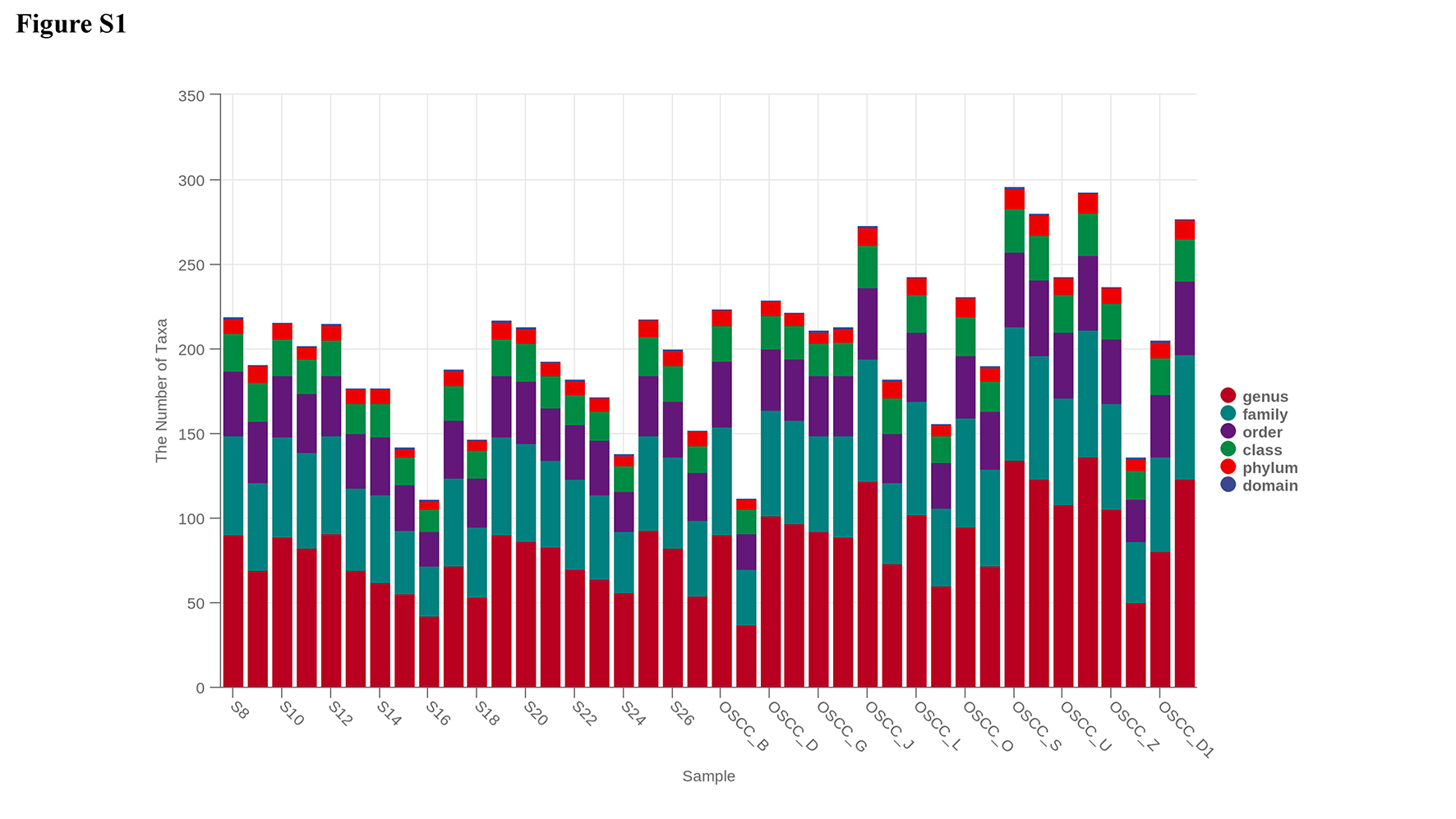

Supplement: Supplementary Figure 1 — Histogram showing the reads that fell into different taxonomic levels of the bacteria by sample. [file Image_1.TIF]

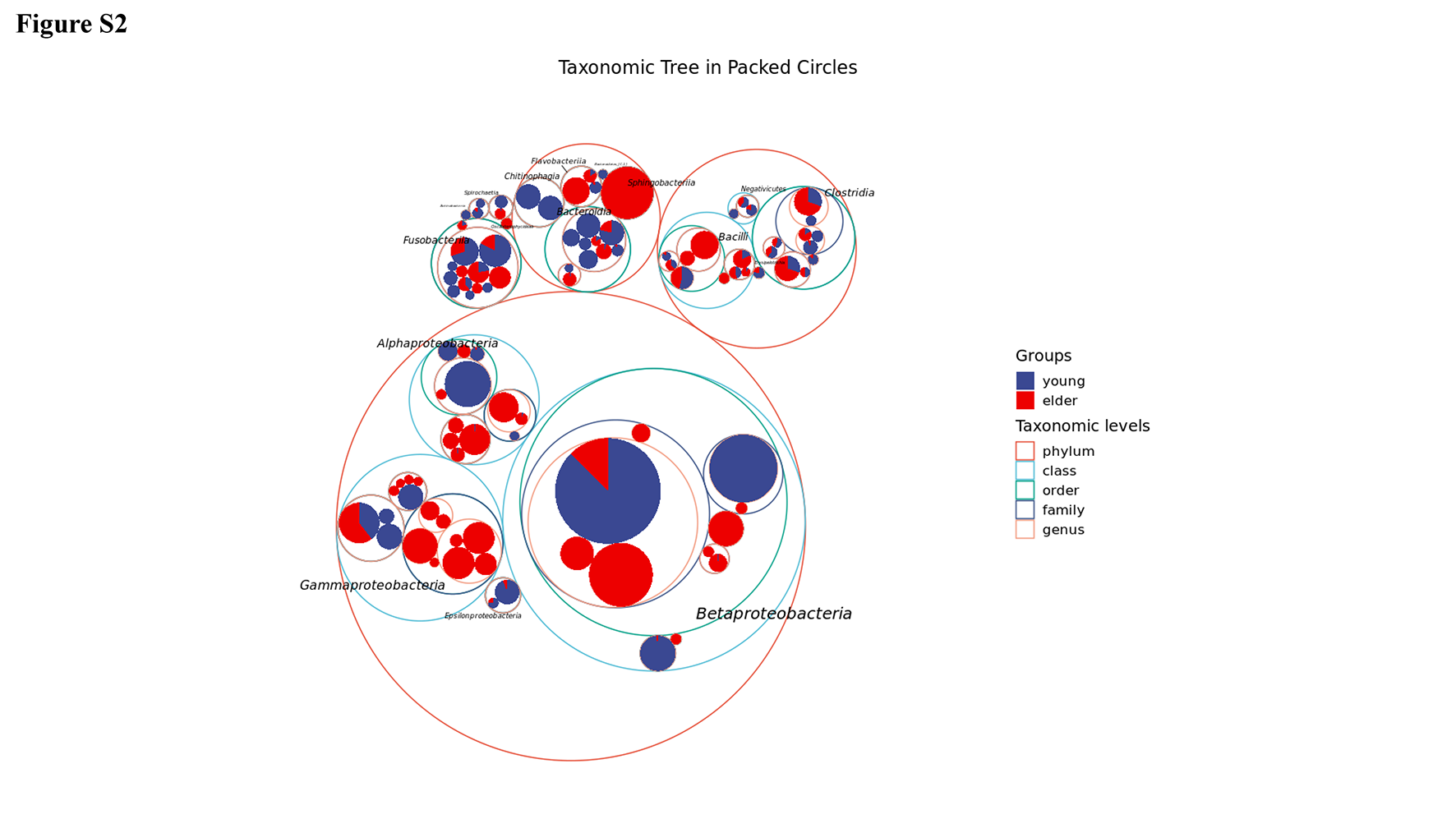

Supplement: Supplementary Figure 2 — Circle packing chart exhibiting the taxonomic tree of microbiota from younger and elder OSCC groups. [file Image_2.TIF]

# Relativegenus and Clinical Index

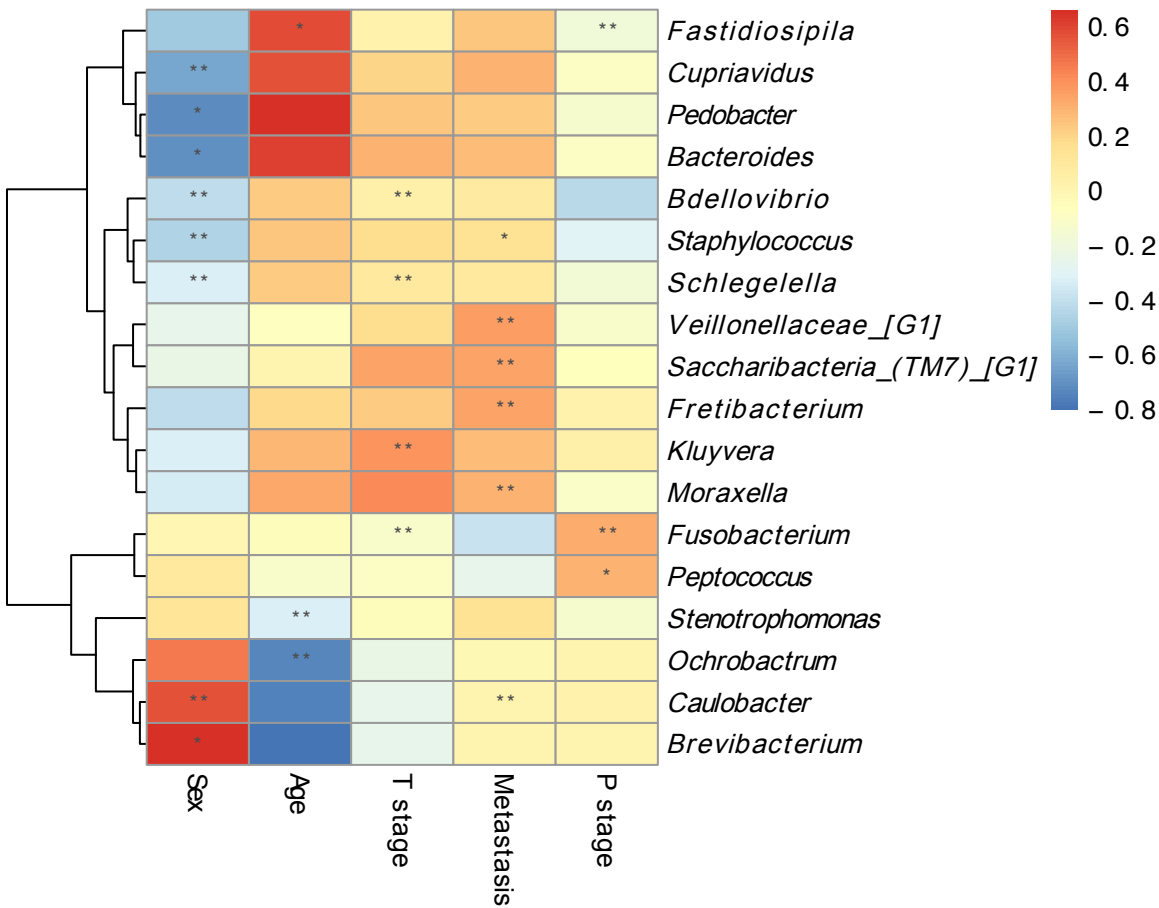

Supplement: Supplementary Figure 3 — Relative analysis of pooled genera from younger and elder OSCC groups with clinical information. [file Image_3.pdf]
